# Dual Targeting of Stromal Cell Support and Leukemic Cell Growth by a Peptidic PKC Inhibitor Shows Effectiveness against B-ALL

**DOI:** 10.3390/ijms21103705

**Published:** 2020-05-25

**Authors:** Paola Fernanda Ruiz-Aparicio, Natalia-Del Pilar Vanegas, Gloria Inés Uribe, Paola Ortiz-Montero, Camila Cadavid-Cortés, Jimmy Lagos, Jessica Flechas-Afanador, Adriana Linares-Ballesteros, Jean-Paul Vernot

**Affiliations:** 1 Grupo de Investigación Fisiología Celular y Molecular, Facultad de Medicina, Universidad Nacional de Colombia, Bogotá D. C. 111321, Colombia; pfruiza@unal.edu.co (P.F.R.-A.); npvanegasa@unal.edu.co (N.-D.P.V.); peortizm@unal.edu.co (P.O.-M.); cacadavidco@unal.edu.co (C.C.-C.); 2 Grupo de Investigación Oncohematología Pediátrica, Fundación Hospital de la Misericordia, Universidad Nacional de Colombia, Bogotá D. C. 111071, Colombia; guribeb@homifundacion.org.co (G.I.U.); jlagosi@homifundacion.org.co (J.L.); jflechasa@unal.edu.co (J.F.-A); talinaresb@unal.edu.co (A.L.-B.); 3 Servicio de Patología, Laboratorio de Hematología Especial y Citometría de flujo, Fundación Hospital de la Misericordia, Bogotá D. C. 111071, Colombia; 4 Instituto de Investigaciones Biomédicas, Facultad de Medicina, Universidad Nacional de Colombia, Bogotá D. C. 111321, Colombia

**Keywords:** leukemic microenvironment, mesenchymal support, PKC, B-ALL, chimeric peptide, cell adhesion

## Abstract

Mesenchymal stem cells (MSC) favour a scenario where leukemic cells survive. The protein kinase C (PKC) is essential to confer MSC support to leukemic cells and may be responsible for the intrinsic leukemic cell growth. Here we have evaluated the capacity of a chimeric peptide (HKPS), directed against classical PKC isoforms, to inhibit leukemic cell growth. HKPS was able to strongly inhibit viability of different leukemic cell lines, while control HK and PS peptides had no effect. Further testing showed that 30% of primary samples from paediatric B-cell acute lymphoblastic leukaemia (B-ALL) were also strongly affected by HKPS. We showed that HKPS disrupted the supportive effect of MSC that promote leukemic cell survival. Interestingly, ICAM-1 and VLA-5 expression increased in MSC during the co-cultures with B-ALL cells, and we found that HKPS inhibited the interaction between MSC and B-ALL cells due to a reduction in the expression of these adhesion molecules. Of note, the susceptibility of B-ALL cells to dexamethasone increased when MSC were treated with HKPS. These results show the relevance of these molecular interactions in the leukemic niche. The use of HKPS may be a new strategy to disrupt intercellular communications, increasing susceptibility to therapy, and at the same time, directly affecting the growth of PKC-dependent leukemic cells.

## 1. Introduction

Bone marrow (BM) stromal cells, in particular mesenchymal stem cells (MSC) play a pivotal role in cellular homeostasis within the BM microenvironment not only in physiological but also in pathological conditions [1]. Cross-talk between MSC and leukemic cells has reciprocal functional consequences [2,3], and also in surrounding cells, with overwhelming consequences [4,5]. The relevance of this interaction for the leukemic cells is evidenced by the fact that despite the upregulation of antiapoptotic genes, primary leukemic cells are in general incapable of surviving ex vivo without a cell stromal support, and need to be co-cultured with for example BM-MSC [6,7]. Importantly, early work has shown that MSC are also responsible for resistance to chemotherapy and disease relapse in different types of leukaemia [8,9,10]. This drug-resistant effect is largely mediated by strong leukemic cell adhesion to stromal cells [11,12]. It is recognized that the interaction between the vascular cell-adhesion molecule-1 (VCAM-1) and the integrins VLA-4 or VLA-5 plays a major role in this cell-to-cell adhesion and chemoresistance [12,13,14,15]. Interestingly, these molecular interactions promoted the activation of the transcription factor NF-κB in MSC, induced by the activation of the classic serine/threonine protein kinase C-βII (PKC-βII) upon leukemic cell binding [16]. Since stromal PKC upregulation was evidenced in various haematological malignancies including chronic lymphoid leukaemia (CLL), acute lymphoid leukaemia (ALL) and mantle cell lymphoma, the authors suggested that this pathway could be a common mechanism of survival in haematological malignancies [16].

The PKC family comprises about 10 different isoforms classified into three groups (classic, novel and atypical) based on their molecular structure and cofactor requirements. They are ubiquitously expressed in tissues and conserved throughout evolution and they regulate different processes during haematopoiesis and immune cell functions [17]. Early work documented the differential enzyme activity of the classical PKC (α, β, γ) in acute myeloid leukaemia (AML), ALL and B type CLL [18]. More recently, it was shown that PKCα activity promotes BCL-2 phosphorylation and chemoresistance in ALL [19], and by inducing RACK1 over-expression PKCα is responsible of T-ALL resistance to vincristine and prednisone [20]. Additionally, PKCα and PKC-βII activity has been associated with cell survival and proliferation in AML and CLL, respectively [21,22,23]. The same authors showed that modulation of PKCα function play a role in CLL development [23,24]. Although classic PKC isoforms have been frequently implicated in leukemic growth, other “novel” isoforms (δ and ε) have also been reported [25].

All these studies in leukaemia and in other different diseases [26] in which PKC has been implicated show the importance of developing PKC inhibitors. This is relevant now that we know that the initial promise of developing small molecules capable of blocking PKC isoforms has been disappointing [26,27]. We have developed a chimeric peptide, HKPS, comprising the hydrophobic portion (HK) of the growth factor from Kaposi’s sarcoma [28] and a short pseudosubstrate sequence (PS) common to all classical PKC isoforms (α, βI, βII and γ). We have previously shown that this chimeric peptide is capable of penetrating cells, strongly inhibiting PKC activity, blocking early cell signalling and proliferation, and being able to inhibit Jurkat (T-ALL) and REH (B-ALL) cell growth [29]. In the present work, we studied the HKPS cytotoxic effect in other leukemic cell lines and in samples obtained from paediatric B-ALL patients. Furthermore, since PKC-βII isoform expression in MSC is essential for leukemic support [6,8,16], we have used the HKPS peptide to weigh the contribution of both, stromal and leukemic cell PKC activity to leukemic cell survival, adhesion and chemotherapy sensitization of B-ALL cells. It should be emphasized that a therapeutic strategy simultaneously targeting the uncontrolled growth of leukemic cells and the support provided by the MSC, constitutes a novel and probably more effective strategy against this disease.

## 2. Results

### 2.1. Cell Growth Inhibition of Leukemic Cell Lines by HKPS

**Figure 1:**
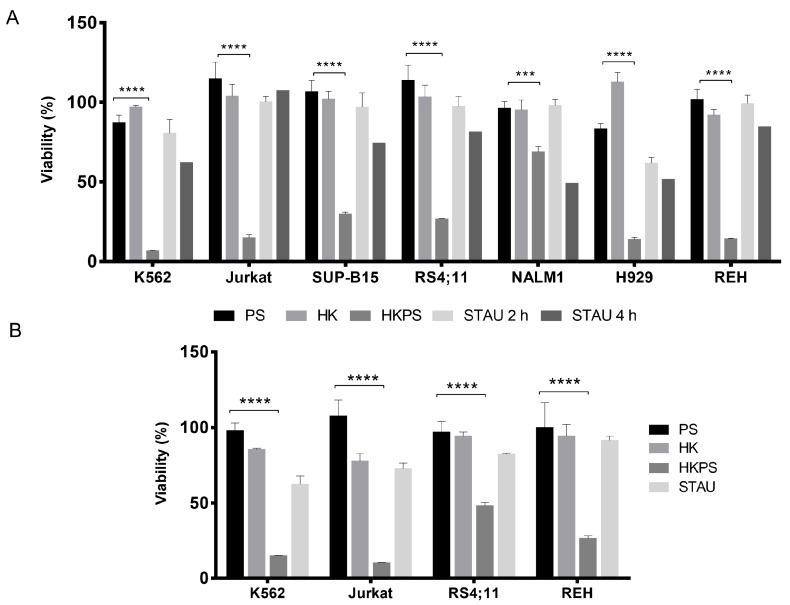
HKPS treatment induces a reduction in leukemic cells viability. (**A**) Leukemic cell lines K562 (CML) JURKAT (T-ALL), SUP-B15 (B-ALL), RS4;11 (B-ALL), NALM1 (CML), H929 (multiple myeloma) and REH (B-ALL), were treated with HKPS (40 μM), HK and PS control peptides (40 μM) for 2 h and STAU (2 μM) for 2 and 4 h. The cell viability was assessed by MTT assay and graphed in relation to the negative control (vehicle). (**B**) A lower (20 μM) HKPS, PS and HK peptide concentration was used in the four different cell lines indicated; 2 μM STAU was used as control. Samples were processed in triplicates in three independent experiments. Data are expressed as mean ± SEM (*p* values: two-way ANOVA *** *p* < 0.001, **** *p* < 0.0001)

Preliminary experiments have shown that the growth of T-lymphocytes and the T-leukemic cell line Jurkat was inhibited by the chimeric peptide HKPS [29] while control peptides HK and PS had no effect. This cytotoxic effect in monocultures was very rapid within 1 h of treatment, and could be observed with concentrations starting from 20 µM. We first sought to know if this cell growth inhibitory effect of HKPS also occurs in other types of leukemic cell lines. Therefore, the peptides (chimeric and controls) were tested in the following leukemic cell lines: REH (B-ALL); SUP-B15 (B-ALL, Ph+); RS4;11 (B-ALL and monocytic characteristics); K562 (CML), Jurkat (clon E61), NALM-1 (CML, Ph+, blastic crisis) and H929 (B-type, multiple myeloma). Except for NALM-1 (25% inhibition), a 2 h treatment with 40 µM HKPS peptide strongly (between 70% and 90%) inhibited the cell growth of the different cell lines (Figure 1A). The control peptides HK and PS did not induce cytotoxicity. The PKC inhibitor Staurosporine (STAU) (1 µM) always showed a reduced effect compared to HKPS, even when treatments were extended to 4 h (Figure 1A). A higher (80 µM) HKPS concentration did not increase the cytotoxic effect seen at 40 µM (not shown), while 20 µM HKPS has still an important inhibitory effect in K562 and Jurkat (>90%), REH (75%) and RS4;11 (50%) cell lines (Figure 1B). 

### 2.2. Cell Growth Inhibition of Leukemic Cells from B-ALL Patients by HKPS 

Since the majority of leukemic cell lines tested were B-type lymphoblast, we were prompted to test the effect of HKPS in primary cells from B-cell precursor ALL patients (Table S1). We chose patients with high blast infiltration (>80%) to be sure that evaluations were done mainly in leukemic cells. B-ALL cells were clearly affected by the chimeric HKPS peptide and the PKC inhibitor STAU as evaluated by light microscopy (Figure S1C). The control peptides HK, PS and HPSscr had no apparent effect. The presence of damaged, opaque and irregular cells was observed at 20 and 40 µM HKPS and 2 µM STAU, although in the former treatments, cells with larger cytoplasm and extracellular debris could be observed; smaller and shrunk cells were observed with 40 µM HKPS (Figure S1C). These results suggested an increased cytotoxic effect of HKPS compared to STAU, as we have already noticed above for the leukemic cell lines.

**Figure 2:**
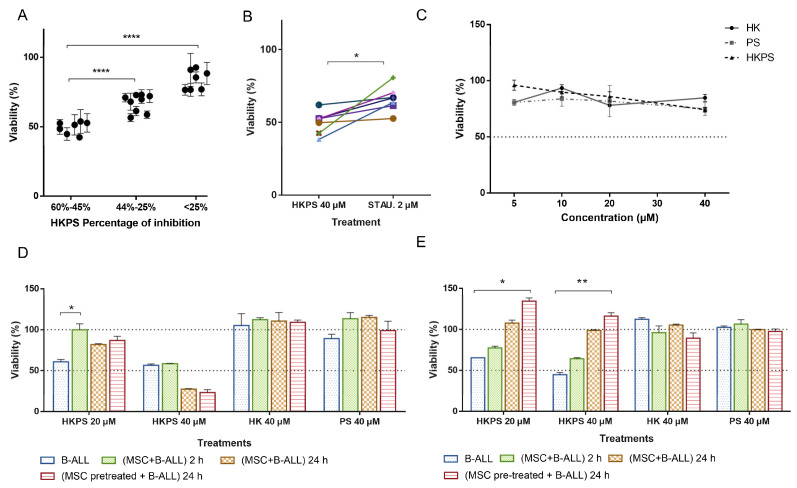
B-ALL patient samples show different susceptibility to HKPS, which was dependent on MSC support. (**A**) According to the susceptibility to HKPS (40 μM, 2 h), B-ALL primary cells (*n* = 23) were classified into three groups. The viability was assessed by the MTT assay. Percentages are expressed relative to B-ALL cells treated with vehicle (DMSO 0.09%). (**B**) Comparative responses in the More HKPS susceptible group to HKPS 40 μM and STAU 2 μM. (**C**) The effect on MSC viability was determined after 2 h of treatment with HK, PS and HKPS at the indicated concentrations by the MTT assay. (**D**,**E**) Representative responses in the more HKPS susceptible group to peptides treatment (20 and 40 μM, as indicated) under the following conditions: B-ALL cells alone for 2 h without support; co-culture of B-ALL cells and MSC for 2 h; co-cultures of B-ALL cells and MSC for 2 h and then cultured for additional 22 h in the presence of 10% FBS; pre-treatment of MSC for 2 h and then co-cultured with untreated B-ALL for additional 22 h. Data are expressed as mean ± SEM (*p* values: ordinary one-way ANOVA (A) Wilcoxon test (B); and non-parametric one-way ANOVA (D,E) * *p* < 0.05. ** *p* < 0.01. **** < 0.0001).

From the 23 B-ALL patient samples tested, seven patients (30.4%) showed higher (> 45%) inhibition at 40 µM HKPS during a single 2 h period treatment; nine patients (39.2%) were not or very low (<25%) affected; seven patients (30,4%) showed an intermediate (45–25%) growth inhibition (Figure 2A). Treatment with 20 µM HKPS showed a reduced effect in all samples in which an important effect was observed at 40 µM (not shown). As with the leukemic cell lines, the control peptides HK and PS did not inhibit B-ALL cell growth. In some patients (*n* = 3), a slightly (about 10–20%) decrease in viability was observed with the HK peptide. The DMSO vehicle at the concentration used for solubilizing the peptides did not produce any effect and this value was used to set 100% cell viability. The STAU positive control produced a variable effect in the B-ALL patient cells, but in the more HKPS susceptible group, it was lower than the effect produced by the chimeric HKPS (Figure 2B). Taking into consideration that STAU is not very specific for the PKC isoforms, and other protein kinases could be affected by this treatment, the higher HKPS effect on B-ALL cells is valuable. A Pearson’s correlation analysis showed a moderate association between the susceptibility to HKPS and the expression of CD13, CD34, CD81, CD24, CD38, the percentage of infiltration of leukemic blasts in the BM at diagnosis and the Minimal Residual Disease (MRD) at day 15 (Figure S2D). Only the correlations with CD9 and CD24 expression were statistically significant (*p* = 0.05). However, the biological relevance of this finding is not completely clear, and these results will require further analysis. 

### 2.3. Cell Growth Inhibition of B-ALL Cells by HKPS in a Co-Culture System with MSC

We also evaluated the effect of HKPS on a co-culture system comprising B-ALL cells and MSC obtained from BM of orthopaedics patients (Figure S2A,B). First, we assayed the effect of peptides on MSC alone. A 2 h treatment with HKPS at low concentrations (5 and 10 µM) was almost without effect (Figure 2C); at 20 and 40 µM HKPS a small/intermediate effect (84–72%) on cell viability was observed, although it was not different from the effect observed with control peptides HK and PS. Prolonged (6 h) exposure of MSC to HKPS slightly affected the viability at lower concentrations (10% at 10 µM and 27% at 20 µM), but at higher concentrations, viability was reduced around 50% (Figure S2C). The HK peptide also induced some cytotoxicity in MSC in this treatment period. A 6 h treatment with STAU induced cytotoxicity (40%) in MSC cells. Therefore, a 2 h treatment with HKPS did not affect significantly affect MSC viability. These effects were very similar in the other MSC samples tested. 

Next, we chose five samples from the more HKPS susceptible group and treated them with the peptides in the following conditions: B-ALL alone (I); B-ALL in the co-culture system (2 h) (II); B-ALL in the co-culture system (24 h) (III); B-ALL in the co-culture system, after HKPS pre-treatment of the MSC (2 h) and further co-culture for 24 h (IV). In general, samples showed two behaviours exemplified in Figure 2D,E: A protective effect of MSC observed at 20 and 40 µM HKPS concentration, which was more evident after 24 h of co-culture (Figure 2D). In this group, B-ALL viability was around 60% (with 20 µM HKPS) and 45% (with 40 µM HKPS), and after 24 h of co-cultivation with MSC it increased to 100%. Pre-treatment of MSC with HKPS for 2 h did not change the protective effect. In the second group, an MSC protective effect could only be observed at 20 µM HKPS, but not at higher (40 µM) HKPS concentrations (Figure 2E). Interestingly, the pre-treatment of MSC with HKPS reduced viability to almost the same values, as the co-cultured cells incubated for 24 h (Figure 2E). These results suggested that MSC protect B-ALL cells from death at lower HKPS concentrations; nevertheless, if the HKPS concentration is higher, the co-culture with MSC is no longer effective at protecting B-ALL cells. Therefore, higher (40 µM) HKPS concentrations could be used to block the supportive effect of MSC and the growth of leukemic cells in some patients. 

**Figure 3:**
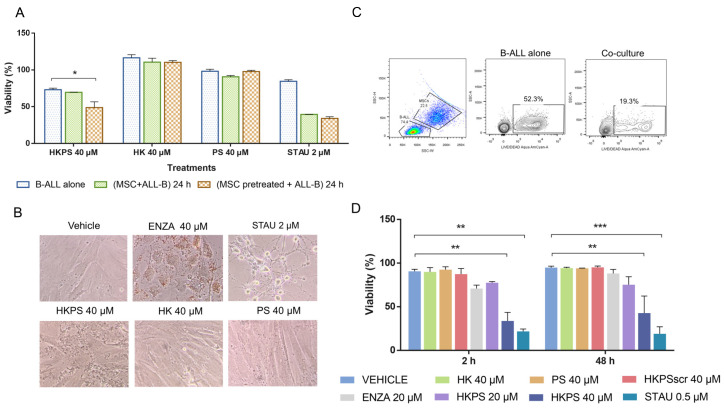
The inhibition of the stromal support by HKPS increases B-ALL leukemic cell death. (**A**) Representative response in the intermediate HKPS susceptible group to peptides treatment (40 μM) and STAU (2 μM) under the following conditions: B-ALL cells alone for 2 h without support; co-cultures of B-ALL cells and MSC for 2 h and then cultured for additional 22 h in the presence of 10% FBS; pre-treatment of MSC for 2 h and then co-cultures with untreated leukemic cells for additional 22 h. Results represent one of three independent experiments. (**B**) Morphological changes of MSC after treatment with HK, PS or HKPS peptides, and ENZA or STAU inhibitors at the indicated concentrations. Representative microphotographs are shown at 20× magnification. (**C**) Effect of MSC support in B-ALL cell viability assessed by LIVE/DEAD Aqua staining by flow cytometry. (**D**) MSC were treated for 2 h or 48 h in the presence of 1% FBS with HKPS, the control peptides and PKC inhibitors ENZA and STAU (20 μM and 0.5 μM, respectively) and then co-cultured for 72 h with primary B-ALL cells. Viability was determined in the leukemic cell population by flow cytometry. Data are expressed as mean ± SEM obtained from two independent experiments (*p* values non-parametric one-way ANOVA * *p* < 0.05. ** *p* < 0.01. *** *p* < 0.001).

Samples (*n* = 3) from the second group with intermediate HKPS susceptibility to HKPS were also studied to evaluate the MSC protective effect. However, in the patient shown in Figure 3A, viability of B-ALL cells alone was reduced to about 75% with HKPS, and this result did not change in the co-culture, in HKPS-pre-treated MSC, viability was reduced to 50%. Control peptides HK and PS did not abolish the protective effect of MSC. The PKC inhibitor STAU affected viability either when treatment was performed in the co-cultures or when MSC were pre-treated (Figure 3A). This is probably due to the fact that STAU (even at lower concentrations) affected strongly MSC morphology (Figure 3B). The effect of HKPS treatment was similar in other two B-ALL samples tested from this group. These results reinforce the protective effect of MSC on B-ALL cell viability, the involvement of classical PKC isoforms in this process and the usefulness of HKPS to inhibit this supportive effect.

### 2.4. Differential Cytotoxicity Evaluation of HKPS in MSC and Leukemic Cells 

As both B-ALL cells and MSC were affected by the chimeric peptide with different intensity, it was important to distinguish HKPS effect in each type of cell in the co-cultures. For this purpose, we used the Aqua Fixable LIVE/DEAD reagent. In the absence of MSC dead B-ALL cells increased up to 52% after 72 h, while in the presence of MSC dead cells were only 19% (Figure 3C). First, we evaluated the effect of the classical PKC inhibitors STAU and Enzastaurine (ENZA) on the RS4;11 leukemic cell line, the latter compound was included since it has been shown to be a more specific inhibitor for the classical PKC isoforms [30]. Although in the leukemic cell lines growth is obviously independent of MSC support, a reduced number of dead RS4;11 cells were revealed when they were co-cultured with MSC (Figure S3A, lower and upper panels, non-treated and vehicle panels). This protective effect was abolished when MSC were treated with the PKC inhibitors ENZA (20 and 40 µM) and STAU (1 µM). Particularly, the treatment with the more specific inhibitor ENZA showed a negative effect on the B-ALL cell viability in a dose-dependent manner. Finally, we compared the Aqua Fixable LIVE/DEAD and the MTT assays. Similar results were obtained with both approaches. However, flow cytometric analysis facilitates also the identification of slightly differences in cell viability in relation to the inhibitor concentration used (Figure S3B).

Thus, the Aqua Fixable LIVE/DEAD assay allowed us to evaluate the MSC support to B-ALL cells under different conditions. Due to the particularities in size and complexity of MSC and B-ALL cells, they can be easily differentiated by flow cytometry. If cell surface markers are used, then the identification is easier and unequivocal, and it was possible, by using the CD19 B-cell surface marker, to be sure that we were evaluating B-ALL cell viability. In patients from the more HKPS susceptible group, this MSC support was lost by the treatment for 2 h of MSC with the PKC inhibitors used, with a higher effect of HKPS 40 µM and STAU 0.5 µM (Figure 3D). Control peptides (HK, PS and HKPSscr) had a minimum effect. According to our previous results, MSC cells were not severely affected by these treatments (Figure S4A). During prolonged treatments (48 h), the effect of these PKC inhibitors was similar (Figure 3D). From these experiments, it is clear that the chimeric peptide HKPS has an important effect on the support provided by the MSC to B-ALL cells. This indirect effect will be more important when B-ALL cells are less affected, as is the case here with lower HKPS concentrations. This weakening of the MSC support, together with the direct effect on B-ALL cell viability described above, showed the effectiveness of this dual strategy.

The above experiments suggested that although PKC is a ubiquitous enzyme, the inhibitory HKPS peptide does not have a cytotoxic effect on any cell and it seems that only leukemic cells that depend on PKC for survival are more affected. In the case of the samples from B-ALL patients, only 30% of the samples were drastically affected by HKPS, while the rest were moderately or not affected at all. Additionally, MSC were only relatively (10–25% of viability) affected by the treatment with HKPS up to 40 µM (Figure 2C); of note, MSC could recover after a few hours of HKPS treatment (Figure S4A). Since we had shown that HKPS inhibited the growth of purified normal MNC [29], we proceeded to evaluate whether MNC were protected by the MSC support. In fact, there was a reduction in cell mortality from 95% to 34% when MSC were present (Figure S4B). To prove this in a more real context, we established co-cultures of PKC inhibitor-pre-treated MSC with a mixture of MNC and REH cells (proportion 1:1). The PKC inhibitors ENZA, HKPS and STAU seem to affect less the viability of the MNC than that of the REH leukemic cells (Figure S4C). MNC in the presence of MSC are fairly protected from HKPS and additionally they are less affected than leukemic cells. This will surely be more evident when using B-ALL patient samples that are more dependent on the mesenchymal support.

### 2.5. Role of PKC Inhibition in Cell Adhesion between MSC and B-ALL Cells

**Figure 4:**
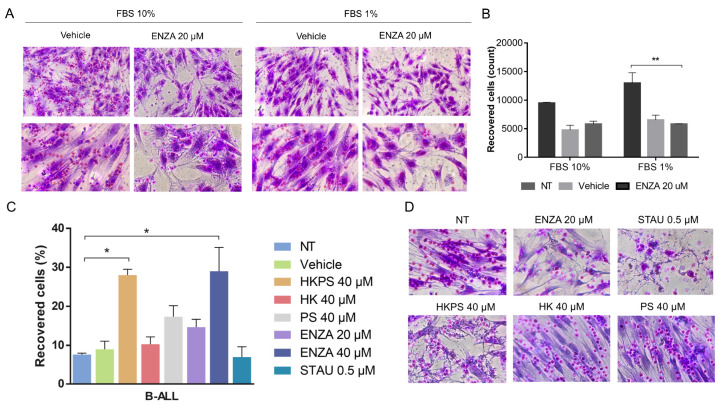
HKPS reduces the adhesion capacity of the B-ALL cells to MSC. (**A**) MSC were treated for 2 h with the PKC inhibitor ENZA or the vehicle (DMSO 0.4%) in co-cultures with B-ALL cells for 6 h at different FBS concentrations. Microphotographs show adherent B-ALL cells to the MSC support stained with crystal violet. Representative microphotographs are shown at 10× and 20× magnification. (**B**) B-ALL cells were recovered by washing with PBS 1× and counted using a hemocytometer. (**C**) MSC were treated as indicated and the percentage of leukemic cells recovered was calculated based on the input of the B-ALL cells and the cells present in the supernatant after washing the co-cultures with PBS 1×. (**D**) Adherent cells in the co-cultures were stained with crystal violet after removing the non-adherent B-ALL cells. Representative microphotographs are shown at 20× magnification. Data are expressed as mean ± SEM (*p* values: nonparametric one-way ANOVA * *p* < 0.05. ** *p* < 0.01).

Since it has been shown that protection from drug treatment is due to cell adhesion [11,12], we then proceeded to assess MSC adhesion of two leukemic cell lines and one sample from the more HKPS susceptible B-ALL patient group. Interestingly, B-ALL primary cells had higher adhesion capacity than leukemic cell lines (Figure S5A), indicating that these cells are probably more dependent on MSC adhesion for survival. To evaluate whether B-ALL cell interaction with MSC was affected by PKC inhibition, we first treated the MSC with ENZA for 48 h in two conditions, 1% or 10% FBS, and showed that B-ALL cell adhesion was highly affected (Figure 4A). Almost twice as many non-adherent B-ALL cells were recovered from the ENZA-treated MSC in both conditions (Figure 4B), although a statistically significant difference was obtained only at 1% FBS. The same B-ALL sample was used to test the chimeric and control peptides and to compare to other PKC inhibitors. In this case, treatments were done for 2 h and in the absence of FBS, after which cells were incubated in culture medium with 1% FBS for the rest of the incubation period (48 h). As can be seen, 40 µM HKPS had the same effect of 40 µM ENZA, while control peptides HK, PS and HKPSscr had no effect (Figure 4C). MSC morphological changes with all PKC inhibitors were evident. Nevertheless, although STAU produced a stronger effect with the presence of damaged cells and of extracellular debris (Figure 4D and Figure S5B), it had no effect on cell adhesion between MSC and B-ALL cells. This result was very similar in another B-ALL sample tested (Figure S5C).

### 2.6. Role of Soluble Factors and Cell–Cell Interactions in MSC Protection

**Figure 5:**
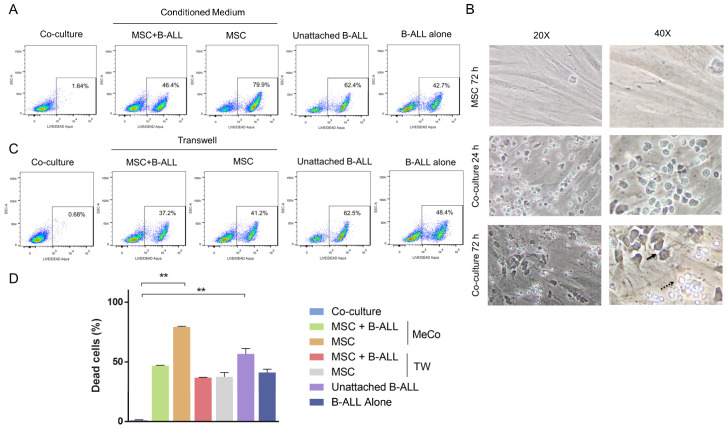
Cell to cell interaction between MSC and leukemic cells is indispensable for B-ALL cell survival. (**A**) B-ALL cells viability was assessed by flow cytometry after exposure for 24 h to fresh conditioned media obtained from a 72 h co-culture of B-ALL and MSC or from a culture of MSC alone. Control of leukemic cells in direct contact with MSC support showed the highest percentage of viability (left panel, Co-culture). Percentages in squares correspond to CD19+ B-ALL cells positive for LIVE/DEAD Aqua staining. The gate was established respect to the control corresponding to the unlabelled co-culture. (**B**) Changes in the appearance in the leukemic cell population were observed in the co-cultures from which the conditioned media were obtained. Unattached leukemic cells looked refringent (dotted arrow) while adherent ones were particularly opaque (solid arrow). Representative microphotographs are shown at the indicated magnifications. (**C**) Survival of B-ALL cells in the presence of soluble factors produced by the co-culture or MSC alone was evaluated in a Transwell system. B-ALL cells alone or B-ALL cells and MSC in direct contact were used as controls. Analysis was done as explained above. (**D**) Quantification of LIVE/DEAD Aqua positive B-ALL cell populations (dead cells) for each of the experiments described in A and C. Data are expressed as mean ± SEM obtained from two independent experiments (*p* values: nonparametric one-way ANOVA. ** *p* < 0.01).

Next, we explored if direct cell–cell contact or soluble factors were responsible for B-ALL cell protection. B-ALL cells were not fully protected by the conditioned media (CM) used, with the CM from the co-cultures producing almost 50% and that from MSC about 80% of dead cells (Figure 5A,D). This modest effect was progressively lost during the next 48 h (Figure S6A,B), suggesting that soluble factors induced in the co-culture contribute to cell survival only during the first 24 h. The majority of unattached B-ALL cells from the co-cultures were already dead (Figure 5A,D, unattached B-ALL cells) reinforcing the idea that direct cell contact between MSC and B-ALL cells is essential for survival: at 24 h, B-ALL cells were attached to MSC and had changed their normal round shape to become more irregular; at 72 h adherent cells looked opaque and many of them lie under the MSC layer while unattached cells were refringent and rounded (Figure 5B).

We also evaluated this issue in TW experiments (Figure 5C). The idea here was to evaluate whether the continuous production of fresh soluble factors could be more effective than the soluble factors produced in a co-culture system during 72 h that are collected and used at the end of this period. Here again, it is shown that soluble factors, even freshly produced, are ineffective in conferring full protection to B-ALL cells (Figure 5C,D). Changes were more evident at 48 h and 72 h in TW co-cultures (Figure S6C,D). These results are equivalent to what we found above with the conditioned media, and they reinforced the argument that cell-to cell interactions are essential for B-ALL cell survival.

### 2.7. Adhesion Molecules Involved in MSC and Leukemic Cell Interactions

**Figure 6:**
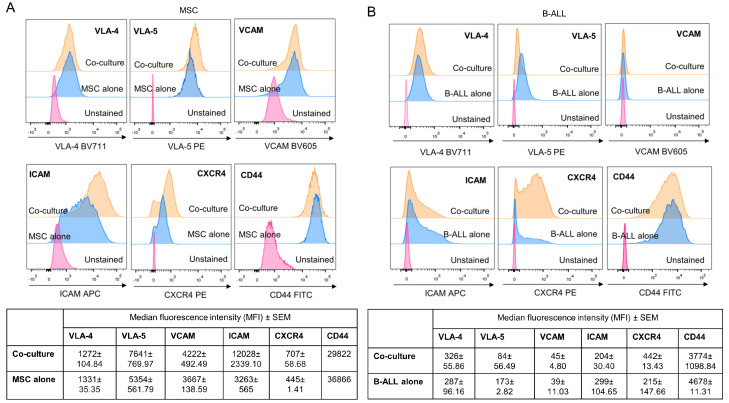
Cell to cell interaction promotes changes in the expression profile of different adhesion molecules in MSC and B-ALL cell populations. (**A**) A representative experiment showing the expression of VLA-4, VLA-5, VCAM, ICAM-1, CXCR4, and CD44 by flow cytometry in MSC alone or in co-cultures with B-ALL cells stablished for 6 h. (**B**) Unattached B-ALL cells and B-ALL cells recovered from the co-culture were labelled for assessing the expression of the indicated molecules. Data are expressed as mean of MFI ± SEM obtained from three independent experiments.

Taking into account that our previous results showed that the treatment with HKPS reduced the adhesion of the B-ALL cells to the MSC support, we next explored some adhesion molecules that could be responsible for this effect in three B-ALL patients. First, we determined the expression of VCAM, VLA-4, VLA-5, ICAM-1, CXCR4 and CD44 both in MSC and B-ALL cells individually and then in the co-culture. We found that MSC expressed all these cell adhesion molecules, and in the co-cultures they increased the expression of VLA-5, ICAM-1 and CXCR4 compared to MSC alone (Figure 6A). The expression of VLA-4, VCAM and CD44 was unaffected (Figure 6A). B-ALL cells expressed VLA-4, VLA-5, ICAM-1 and CD44, low levels of CXCR4 and no expression of VCAM (Figure 6B). In the co-cultures, expression of VLA-5 increased in 2 out of 3 patients or unmodified in one patient, while CXCR4 increased in the three patients studied; ICAM-1 and VLA-4 expression was reduced (Figure 6B). CD44 expression was not modified.

**Figure 7:**
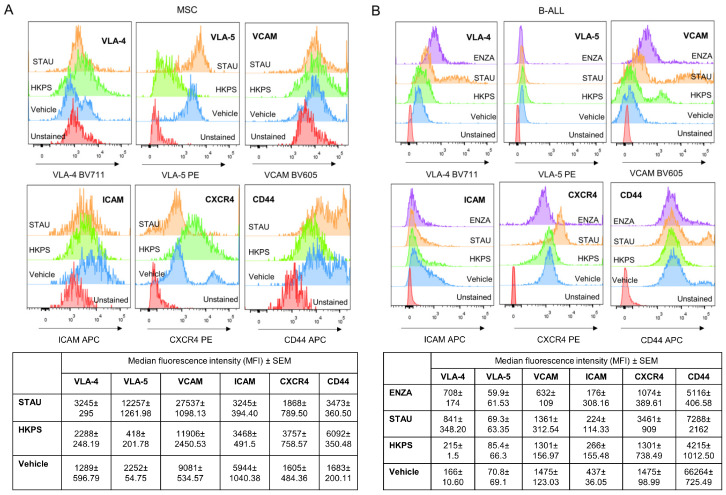
HKPS treatment reduces the expression of molecules involved in the MSC and B-ALL interaction. (**A**) MSC were pre-treated for 2 h with HKPS (40 μM), STAU (0.5 μM) or vehicle (DMSO 0.4%) and then co-cultures with B-ALL cells were stablished for 6 h. A representative experiment showing the expression of VLA-4, VLA-5, VCAM, ICAM-1, CXCR4, and CD44 evaluated by flow cytometry in CD105+ cell population. MSC alone were used as control. (**B**) Unattached B-ALL cells and B-ALL cells recovered after trypsinization from the co-cultures with MSC were labelled for assessing the expression of the indicated molecules in the CD19+ cell population. Data are expressed as mean of MFI ± SEM obtained from two independent experiments.

In the co-cultures, treatment of MSC with the HKPS peptide induced a decrease in the expression of CD44; in particular, a high CD44-expressing cell population was not observed (Figure 7A). Interestingly, VLA-5 and ICAM-1 expression was diminished or abolished by HKPS treatment in MSC and to a lesser extent in B-ALL. Neither of these changes were observed with the STAU inhibitor; ENZA treatment produced MSC auto-fluorescence in some channels, and therefore some molecules couldn’t be evaluated. On the contrary, CXCR4 expression that was already upregulated in the co-culture in both B-ALL cells and MSC was further upregulated by HKPS treatment of MSC and in B-ALL cells, although to a lesser extent in the latter (Figure 7A). Here again this result was not observed with STAU. VCAM and VLA-4 expression was not significantly affected by HKPS (Figure 7B).

In summary, important cell adhesion molecules (ICAM-1 and VLA-5) whose expression was upregulated in the co-cultures were down regulated by HKPS treatment of MSC, suggesting that they may be responsible for the low adhesion observed with the HKPS treatment and consequently the increased susceptibility of B-ALL to this peptide. Interestingly, CXCR4 expression was upregulated in both MSC and B-ALL cells in the co-culture and was further upregulated by the HKPS treatment.

### 2.8. HKPS Sensitization of MSC to Dexamethasone Treatment

To test whether the HKPS peptide could be a complementary treatment to the conventional ones used in leukaemia, we pre-treated MSC with the HKPS peptide and then studied the susceptibility of B-ALL cells to dexamethasone (DEXA) in two patients from the HKPS most susceptible group. A preliminary evaluation in the leukemic cell line RS4;11 and in three different B-ALL patient samples allowed us to determine the appropriated chemotherapeutic agent concentration needed for the treatment of the leukemic cells alone for a short period of time (Figure S7). This was relevant, considering that B-ALL primary cells require the MSC support for survival. We established that cell viability in three B-ALL patients’ samples was reduced to about 50% with DEXA treatment at concentrations equal or higher than 125 nM for 6 h (Figure S7A). In the case of the RSA4;11 cell line, we established that 250 nM DEXA did not affect the cell viability after 6 h of treatment but, if DEXA was removed and the cell culture was maintained for additional 42 h, cell viability was reduced below 50% (Figure S7B). B-ALL cells exhibited a higher susceptibility (50% of viability) to DEXA compared to RS4;11 cells after 6 h of treatment (100% of viability) (Compare Figure S7B and S7A). In this way, the role of the MSC support could be evaluated.

**Figure 8:**
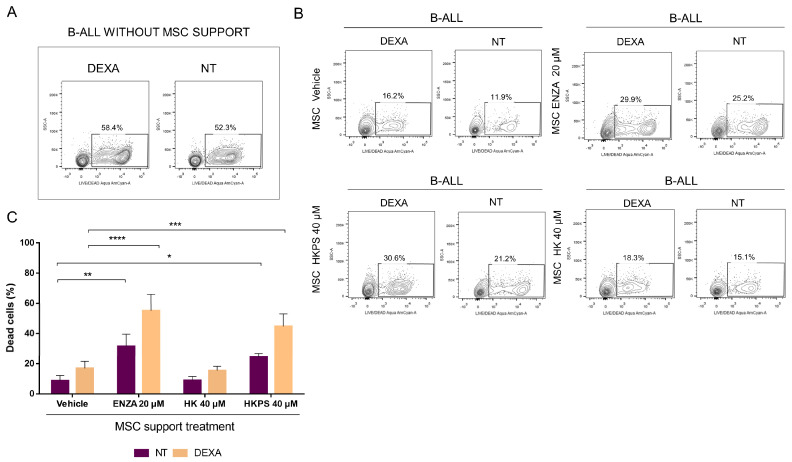
HKPS treatment weakens the MSC support and confers a greater susceptibility to DEXA treatment in B-ALL cells. (**A**) Viability of B-ALL cells treated or not with DEXA for 6 h in the absence of the MSC support was evaluated by flow cytometry. (**B**) MSC were pre-treated for 2 h with HKPS, HK peptides (40 μM) ENZA (20 µM) or vehicle. B-ALL cells exposed or not (NT) to DEXA (250 nM) for 6 h were co-cultured with the previously treated MSC. The viability of B-ALL cells was evaluated by flow cytometry 72 h after establishment of the co-cultures. Percentages in squares correspond to CD19+ B-ALL cells positive for LIVE/DEAD Aqua staining. The gate was defined with the unlabelled co-cultures and represents one of four independent experiments. (**C**) Quantification of LIVE/DEAD Aqua Positive B-ALL cell populations (*n* = 4) after treatment of MSC support as indicated. Data are expressed as mean ± SEM (*p* values: two-way ANOVA. * *p* < 0.05. ** *p* < 0.01. *** *p* < 0.001, **** < 0.0001).

B-ALL cells died very quickly without MSC support, almost similar to DEXA-treated B-ALL cells (Figure 8A). As expected, co-culturing B-ALL cells with MSC for 72 h after leukemic cell treatment was able to rescue the leukemic cells from death (Figure 8B,C, MSC vehicle). A treatment of MSC with 40 µM HKPS or 20 µM ENZA for 2 h showed an increased in cell death in DEXA-treated B-ALL cells (Figure 8B,C). The control HK peptide had almost the same effect as the vehicle alone. When treatment was extended to 48 h, no differences were observed between B-ALL cells that were treated or not with DEXA in MSC previously treated with the vehicle, ENZA or the HK peptide (Figure S7C); meanwhile, in the HKPS-treated MSC, DEXA induced almost three times more B-ALL cell death (Figure S7C). The effect of HKPS on MSC and the increased susceptibility to DEXA were very similar in the other patient’s samples (Figure 8C). These results showed that HKPS has a prolonged inhibitory effect on MSC support compared to other PKC inhibitors, without affecting MSC viability and that susceptibility to conventional chemotherapy can be increased by this mean.

## 3. Discussion

The overall prognosis of paediatric B-ALL patients has improved in the last decades, with 80%-90% patient survival in developed countries. Nevertheless, relapse due to leukemic cells able to evade chemotherapy is still a problem in adult and childhood B-ALL patients [31,32]. Cell adhesion between stromal cells of the BM microenvironment and leukemic cells is required for leukemic survival and contributes importantly to drug resistance [8,33,34]. Novel approaches are necessary for complete eradication of resistant and aggressive cells; in particular, sensitization to chemotherapy is relevant to extend survival of B-ALL patients. Since BM is the site of first relapse in the majority of childhood B-ALL [35], knowing the contribution and mechanisms of stromal cells protection is critical to these novel strategies.

PKC is rarely mutated in haematological malignancies. Nevertheless, it has been shown that its associated signalling is critical for leukemic cell growth and disease progression [21,22,23,24] and also for chemoresistance [19,20,25]. In fact, we found that very dissimilar leukemic cell lines (B-ALL, T-ALL, CML, multiple myeloma), were severely affected by a 2 h treatment with 40 µM HKPS, a chimeric peptide able to penetrate cells and inhibit PKC activity [29]. Of note, growth inhibition of leukemic cell lines by HKPS was stronger than the one attained with the classical PKC inhibitor STAU, known to affect also other protein kinases [36,37,38].

These results prompted us to test this peptidic chimera in BM cell samples from paediatric B-ALL patients. We have shown here that HKPS was able to affect significantly leukemic cell viability in about 30% of B-ALL paediatric patients: between 60% and 45% reduction in cell viability was obtained with 40 µM HKPS. A significant proportion (39%) of other B-ALL patients showed an intermediate to low (44–25%) susceptibility to HKPS. These results suggested that independently from the genetic defect responsible of directing leukemic cell growth in B-ALL patients, PKC activity and its corresponding signalling participate in B-ALL cell survival, although differentially. Finally, 30% of the B-ALL patient cells studied were unaffected by the HKPS treatment and other signalling pathways must be considered to account for cell survival in this group. Control peptides HK, PS and HKPSscr were almost without effect. The fact that the chimeric peptide with the PS scrambled sequence (HKPSscr) did not have an effect in the assays used confirmed the specificity of the functional PS sequence. Interestingly, HKPS has an increased or equivalent cytotoxic effect on B-ALL cells than the STAU and ENZA inhibitors.

PKC-β is believed to be the main isoform mediating NF-κB activation in normal B cells [39], a transcription factor strongly associated with integrins and cell adhesion molecules signalling, among others [12]. The fact that PKC-βII upregulation in stromal cell was observed after leukemic cell binding, and that its expression is necessary for leukemic cell growth and survival [16], provides an exceptional opportunity to use HKPS to hit both leukemic cells and their stromal cell support. We have shown here that co-cultures with MSC increased survival of B-ALL cells, and that this pro-survival effect was diminished by PKC inhibition with HKPS. The effect was not identical in the patients’ samples tested, and some of them required higher concentration of HKPS. Importantly, MSC treatment with HKPS for 2 h induced the highest B-ALL cell death, and notably, the number of dead cells increased after further incubation for 48 h. On the contrary, MSC treatments with ENZA or STAU reduced the number of B-ALL dead cells in the same period of incubation. Therefore, prolonged HKPS treatment of MSC has a persistent deleterious effect on B-ALL cells compared to the other PKC inhibitors. From these experiments it is clear that the main cytotoxic effect attained in B-ALL cells by impairment of the MSC support was achieved by the HKPS peptide. As already shown above, HKPS adverse effect on MSC was sustained, but they seem to recover easily after treatment. Interestingly, MNC in the presence of the MSC support were less affected than the leukemic cells, suggesting a specific cytotoxic effect towards leukemic cells.

The interactions between stromal cells and leukemic cells through adhesion molecules are complex [40]. VLA-4 and VLA-5 mediate the adhesion of B-ALL leukemic cells to stroma [6] and also exert an important role in the homing and engraftment of the leukemic cells [40,41]. VCAM-1 and fibronectin in MSC are playing a central role as ligands for VLA-4 and VLA-5 on ALL cells [42,43]. In addition, B-ALL cell lines have a differential adhesion capacity to the stromal support, with the most adherent ones displaying low sensibility to chemotherapeutic agents [44,45]. Here, HKPS treatment of MSC induced a detachment of B-ALL cells from the MSC with almost 50% of the B-ALL cells found in the culture supernatant. Of note, while STAU seems to affect MSC morphology drastically, it did not produce a major effect on B-ALL cell binding. ENZA and HKPS, affecting less MSC morphology, had a high and similar effect on B-ALL cell adhesion. We therefore suggest that this adhesion inhibition is responsible for B-ALL cell susceptibility, based mainly on two facts: 1) We have shown that the soluble factors produced by MSC or by the co-cultures were unable to protect B-ALL cells from death; and 2) some of the adhesion molecules (VLA-5 and ICAM-1) expressed in MSC that have been implicated normally in interactions between MSC and both HSC and leukemic cells [46,47,48], and which we have shown to be upregulated during co-cultures with B-ALL cells, were importantly down-regulated by HKPS. Interestingly, we found a significant correlation between the HKPS susceptibility and the CD9 expression, a molecule that has been recently proposed as new prognostic marker [49]. CD9 directly interacts with adhesion molecules, enhancing the B-ALL cell association with BM stroma. Of note, the blocking of CD9 was also associated with enhanced chemosensitivity in stromal cell co-cultures [49].

MSC in other leukaemia models had also been associated with leukemic cells survival during chemotherapy [50]; this is due in part to migration of leukemic cells beneath MSC, a phenomenon that has been associated with the expression of functional CXCR4 [51]. Interestingly, we have shown that SDF-1 expression and secretion in MSC is reduced in an in vitro model of the leukemic niche [52], explaining the observed increase in CXCR4 expression in B-ALL cells during the co-cultures. If this effect is further reinforced by HKPS, as we have shown here, then B-ALL cells will be more prone to detach from MSC.

The signalling associated with these adhesion molecules is unknown in B-ALL. In CLL it has been reported that targeting cell adhesion sensitized leukemic cells to chemotherapeutics and inhibition of PKC or PI3K overcome the stroma-induced resistance [53]. We showed here that B-ALL cells treated with DEXA were much more susceptible to treatment when they were co-cultured with HKPS-treated MSC. This reinforces the concept that directing the drug arsenal against both MSC support and the leukemic cells is a more effective treatment for this disease.

Although interfering with cell interactions by blocking adhesion molecules has been shown to be a promising approach to overcome drug resistance [54,55], high variability in adhesion molecule expression in ALL and other leukaemias was observed [33,56]. Despite these differences in ALL cell adhesion phenotypes, stromal cells are invariably capable of protecting leukemic cells. Therefore, only few intracellular signalling pathways should be involved in protection or alternatively several pathways converge on few specific signalling nodes. This seems to be the case here, where we showed that all B-ALL patients tested for adhesion were susceptible to HKPS treatment.

Finally, it has been shown that tumour microenvironment signals are responsible for reduced treatment efficacy in other cancer cells [57,58,59]. In colon carcinoma, it was shown that sites that give Adriamycin resistance had elevated levels of PKC [60]. This suggests that our findings regarding B-ALL treatment with specific PKC inhibitors may have a more widespread application than previously thought.

## 4. Materials and Methods

### 4.1. BM-MSC Isolation (Stromal Cell Support)

MSC were isolated from healthy paediatrics donors who consulted for traumatic events (Fundación Hospital de la Misericordia, HOMI) after the authorization of their parents, who signed the informed consent. The Ethical Committees of the participant institutions: Faculty of Medicine, Universidad Nacional de Colombia and HOMI approved the protocols (007-080-17, 11 May 2017). Femoral BM samples were collected in a sterile tube containing 0.25% EDTA in PBS 1× (GIBCO-Invitrogen, Gran Island, NY, USA). Mononuclear cells were isolated by Ficoll-Hypaque density gradient centrifugation (Histopaque d= 1.077 g/mL, Sigma-Aldrich, St. Louis, MO, USA) and plated at a density of 10^6^ cells/mL in Iscove’s modified Dulbecco’s medium (IMDM) supplemented with 1% sodium pyruvate (GIBCO-Life Technologies, Grand Island, NY, USA), 1% minimum essential medium (MEM), non-essential amino acid solution 100× (GIBCO-Life Technologies, Grand Island, NY, USA), and 10% foetal bovine serum (FBS) (GIBCO-Life Technologies, Grand Island, NY, USA). When MSC had reached confluence (90%), cells were trypsinized with 0.25% Trypsin (Sigma-Aldrich, St. Louis, MO, USA) and 1 mM EDTA, and characterized by immunophenotyping and multipotent differentiation capacity assays [52]. For the different experiments, cells were used in passages 3–6. Ethical aspects regarding human experimentation followed the Declaration of Helsinki principles [61].

### 4.2. Immunophenotypic and Multipotent Differentiation Capacity in BM-MSC

The following monoclonal antibodies were used for MSC immunophenotypic characterization (Figure S2A): FITC mouse anti-human CD73 (clone AD2, BD Pharmingen, San Jose, CA, USA), APC mouse anti-human CD105 (clone SN6, Invitrogen, Frederick, MD, USA), FITC mouse anti-human CD90 (clone F15-42-1, Abcam, Cambridge, MA, USA), and FITC anti-human CD44 (clone MEM-85, Invitrogen, Frederick, MD, USA). Absence of the haematopoietic markers CD45 and CD34 was evaluated with leucocyte-specific antibody PerCP mouse anti-human CD45 (clone 2D1, BD Biosciences, San Jose, CA, USA) and the APC mouse anti-human CD34 (clone 581, BD Pharmingen, San Jose, CA, USA). Data were acquired using a FACSAria IIIup flow cytometer (Becton Dickinson Biosciences, San Jose, CA, USA) and the FACSDiva and FlowJo software (Becton Dickinson Biosciences, Sunnyvale, CA, USA) were used for data analysis [52].

Multipotent differentiation capacity was evaluated following ISCT criteria, using the StemPro Kit for osteo-, adipo- and chondrogenic differentiation as previously reported [62]. Briefly, for osteogenic differentiation induction, MSC were cultured for 21 days with StemPro Osteogenesis Differentiation Kit and for adipogenic and chondrogenic differentiations, MSC were cultured for 14 days with StemPro Adipogenic and StemPro Chondrogenic Differentiation Kit (Thermo Fisher Scientific Inc, GIBCO by Life Technologies, Waltham, MA USA), respectively. For staining, cells were fixed with 4% formaldehyde (Sigma-Aldrich, St. Louis, MO, USA), and stained with 0.35% Oil Red O solution (Sigma-Aldrich, St. Louis, MO, USA) for adipogenic differentiation or with an NBT/BCIP Colour Development Substrate Kit (Promega, Madison, WI, USA) for osteogenic differentiation or with 0.1% Safranin O (Sigma-Aldrich, St. Louis, MO, USA) for chondrogenic differentiation [52]. Cells were examined with an inverted microscope (Eclipse Model TS-100, Nikon, Konan, Minato-ku, Tokyo, Japan), photographed with a PowerShot A460 camera and using the Zoom Browser EX software (Canon, Melville, NY, USA). Once characterized, MSC were frozen in multiple aliquots for later use. MSC were thawed and cultivated by standard methods and aliquots from the same patients were used for the same type of experiments (a representative differentiation assay is shown in Figure S2B).

### 4.3. Immortalized Leukemic Cell Lines

The human REH (ATCC CRL-8286, American Tissue Culture Collection, Rockville, MD, USA), SUP-B15 (ATCC CRL-1929), RS4;11 (ATCC CRL-1873), NALM-1 (ATCC CRL-1567), Jurkat (ATCC TIB-152), K-562 (ATCC CCL-243) and H929 (ATCC CRL-9068) cell lines, were kept at 37 °C and 5% CO2 in complete medium (RPMI 1640 or IMDM supplemented with 1% sodium pyruvate, 1% non-essential amino acids and 10% FBS).

### 4.4. Patient B-ALL Cells Isolation and Characterization

Paediatric patients with Type-B Acute Lymphoblastic Leukaemia (B-ALL) (*n*= 23) were diagnosed at the Fundación Hospital de la Misericordia (HOMI), in the Paediatric Oncohaematology Unit. The diagnosis was established according to the WHO criteria. Immunophenotype was achieved by flow cytometry (FACScanto II, Becton Dickinson Biosciences, San Jose, CA, USA) using the Euroflow panels ALOT (cyCD3-v450, CD45-V500, cyMPO-FITC, cyCD79aPE, CD34-PerCPCy5.5, CD19-PECy7, CD7-APC, CD3-APCH7); Tube 1B (CD20-V450, CD45-V500c, CD58-FITC, CD66c-PE, CD34-PerCPCy5.5, CD19-PECy7, CD10-APC, CD38-APCH7); Tube 2B (smIgk-V450, CD45-V500c, cyIgM-FITC, CD33-PE, CD34-PerCPCy5.5, CD19-PECy7, sIgM/CD17-APC, smIgλ-APCH7); Tube 3B (CD9-PB/V450, CD45-V500c, TdT-FITC, CD13-PE, CD34-PerCPCy5.5, CD19-PECy7, CD22-APC, CD24-APCH7) and Tube 4B (CD21-V450, CD45-V500c, CD34-PerCPCy5.5, CD19-PECy7, CD123-APC, CD15-FITC, CD81-APCH7), following the recommendations for lineage assessment (www.euroflow.org). Data were analysed using the Infinicyt Software, v. 2.0 (Cytognos SL, Salamanca, Spain). Samples collected from the patients who accepted to participate in this study and whose parents have agreed to sign the informed consent were used in this study. The Ethical Committees of the Faculty of Medicine, Universidad Nacional de Colombia and of the Fundación Hospital de la Misericordia approved the protocols (007-080-17, 11 May 2017). Human BM blood mononuclear cells were isolated from newly diagnosed B-ALL patients by a Ficoll-Hypaque gradient centrifugation as previously described. Only BM samples with a high (>80%) leukemic blasts infiltration were used. Ethical aspects regarding human experimentation followed the Declaration of Helsinki principles [61].

### 4.5. Establishment of the Co-Cultures of Leukemic Cells with MSC

5 × 10^4^ B-ALL patient or leukemic cell line cells were seeded on a layer of cultured MSC at a confluence of 70–100% (depending on the experiment). As controls, B-ALL cells or MSC alone (in some experiments) were seeded in RPMI-1640 medium. The cultures were maintained for 24, 48 or 72 h.

### 4.6. Peptide Synthesis and Characterization

Three peptides were used and synthesized using the SPPS-Fmoc/tBu methodology, as previously reported [63]: 1) The sequence corresponding to the hydrophobic signal from the fibroblast growth factor derived from Kaposi’s sarcoma, AAVALLPAVLLALLAP, here on called HK [28], 2) The pseudosubstrate sequence (hereafter called PS) RKGALRQY, common to the four classical PKC-isoforms [18,64] and 3) the chimeric HKPS resulting from coupling HK and PS with the sequence AAVALLPAVLLALLAPRKGALRQY [29]. In some experiments, we used as control a scrambled sequence of PS coupled to the HK sequence (the chimeric HKPSscr peptide). Peptides were purified by RP-HPLC and characterized by MALDI-TOF mass spectrometry. Peptides with >90% purity were used (Figure S1A).

### 4.7. Cytotoxicity of Leukemic Cells Induced by PKC Inhibitors

The cytotoxic effect of peptides and PKC inhibitors was first evaluated spectrophotometrically using the MTT assay [65]. For leukemic cell lines, cells were seeded at a density of 5 X 10^4^ cells/100 µL and treatment with peptides was done for 2 h at different concentrations (20, 40 and 80 µM). Staurosporine (STAU) treatment was also performed for 2 and 4 h. In the case of B-cell ALL samples, several evaluations were made: 1) 5 × 10^4^ B-ALL patient cells were treated for 2 h with different concentrations of the HK, PS and HKPS peptides (20 and 40 µM) and subsequently cytotoxicity was evaluated; 2) B-ALL cells were seeded in 96-well microplates containing confluent MSC, and after 2 or 24 h of incubation, the cytotoxicity was determined as described above; 3) In another series of experiments, cytotoxicity was evaluated in the following conditions: MSC and B-ALL cells without treatment; non-treated MSC and pre-treated B-ALL cells; and pre-treated MSC and non-treated B-ALL cells). In some experiments in which the MSC support was evaluated, we used also the HKPSscr peptide. The MTT reagent was added to a final concentration of 50 µg/mL and incubated for 4 h for the evaluation of MSC alone. In preliminary experiments, it was found that primary B-ALL cells were unable to form Formazan crystals after 4 h of MTT incubation (Figure S1B); therefore, we extended the incubation time to 18 h in this case and in the co-cultures. Then, 100 µL of DMSO were added for 15 min to solubilize the crystals. Subsequently, the absorbance at 550 nm was determined in a spectrophotometer (Ultramark, Bio-Rad, CA, USA). As a positive control STAU 2 µM was used. Negative controls included growing cells without any treatment or cells with vehicle alone (DMSO at the concentrations used for dissolving the different compounds).

### 4.8. Differential Cytotoxicity in MSC and Leukemic Cells Determined by Flow Cytometry

The viability of the leukemic cells was also evaluated with the LIVE/DEAD Fixable Aqua Dead Cell Stain (Molecular Probes, Eugene, OR, USA). After incubation of MSC with the PKC inhibitors, the co-cultures were stablished for the indicated time-periods and then double labelled with CD19 FITC (clone HIB19, BD Pharmingen, San Jose, CA, USA) and the LIVE/DEAD Aqua stain for viability evaluation by flow cytometry (FACSAria IIIup Becton Dickinson Biosciences, San Jose, CA, USA); data were analyzed with the FlowJo software. First, the leukemic cell line RS4;11 was evaluated alone or in the co-culture with MSC pre-treated or not with the PKC inhibitors STAU and ENZA. Then, evaluations were performed in B-ALL cells from patients, as follows: 5 × 10^4^ B-ALL cells were seeded in 96-well microplates containing confluent MSC that have been previously treated with the PKC inhibitors or peptides. MSC viability was also evaluated in the co-cultures by labelling with LIVE/DEAD Aqua and CD105 and non-treated MSC were used as controls. Cell viability was determined either after 2 or 48 h of incubation as described above.

### 4.9. Functional Cell Adhesion Assay of Leukemic Cells

Adhesion of leukemic cells to MSC was done after treatment of the latter with 20 or 40 µM HKPS, HK, PS and HKPSscr peptides, or the STAU and ENZA inhibitors for 2 or 48 h. 5 × 10^4^ leukemic cells (REH and SUP-B15 cell lines or B-ALL cells from patients, *n*= 3) were added to MSC and after 6 h of incubation, cultures were washed with PBS 1× and loosely attached cells from each treatment were collected and counted with a Neubauer chamber. Then, cultures were stained with crystal violet (0.5%) and photographed. The percentage of leukemic cells adhered to MSC was calculated based on the input of the leukemic cells and the cells recovered in the supernatant after treatments, or by dissolving the staining with 0.1% EDTA in PBS 1× and reading in a spectrophotometer (Ultramark, Bio-Rad, CA, USA) at 550 nm.

### 4.10. Contribution of Soluble Factors vs. Direct Cell Contact to the MSC Support

#### 4.10.1. Conditioned Media Evaluation

The conditioned media were obtained after co-cultures of MSC with B-ALL cells or MSC alone for 72 h. 1 × 10^5^ MSC/mL were seeded in a culture flask in IMDM complete medium. After 24 h, the MSC were washed with RPMI incomplete medium and 2 × 10^6^ leukemic cells were added on the MSC monolayer in RPMI medium supplemented with 5% FBS. Three days later, the supernatant was collected, centrifuged at 700 g for 5 min to remove unattached B-ALL, and finally filtered using a 0.20 µM filter (Corning Incorporated, Corning, NY, USA). For viability experiments, the B-ALL cells were resuspended in the fresh conditioned media for 72h; at 36 h a re-feeding with new fresh conditioned medium was made. B-ALL cells alone or MSC/B-ALL co-cultures in RPMI-1640 and 5% FBS were included as controls. B-ALL cell viability was assessed by flow cytometry using LIVE/DEAD Aqua staining at 24, 48 and 72 h.

#### 4.10.2. TW Assay Evaluation

For Transwell experiments MSC and B-ALL cells were seeded in the upper chamber of the insert (5 μm pore size, 6.5 mm diameter, Corning Incorporated, Corning, NY, USA) at densities of 1 × 10^4^ and 5 x10^4^ cells, respectively. In parallel, only MSC were seeded in the upper chamber of the insert. The lower chamber was filled with 600 μL of RPMI-1640 supplemented with 5% FBS containing 1.5 x 10^4^ B-ALL patient cells. B-ALL cells alone or MSC/B-ALL co-cultures in the lower chamber were included as controls. The viability of B-ALL cells was assessed at 24, 48 and 72 h by flow cytometry as described above.

### 4.11. Expression of Adhesion Molecules in MSC and B-ALL Cells

After pre-treatment of MSC with the peptides or PKC inhibitors (2 h), co-cultures with B-ALL cells were established for 6 h, after which unattached B-ALL cells were removed by washing with PBS 1×. Then co-cultures were harvested by trypsin/EDTA treatment and cells were stained with monoclonal antibodies and analysed by flow cytometry. MSC and B-ALL cells were identified by staining with APC mouse anti-human CD105 (clone SN6, Invitrogen, Frederick, MD, USA) and with the APC-H7- mouse anti-human CD19 (clone SJ25C1, BD Pharmingen, San Jose, CA, USA) or FITC-mouse anti-human CD19 (clone HIB19, BD Pharmingen, San Jose, CA, USA) respectively. The following monoclonal antibodies were used for staining MSC and B-ALL cells, as previously described [52]: PE mouse anti-human CD49e (clone IIA1, BD Pharmingen, San Jose, CA, USA), APC mouse anti-human CD49d (clone 9F10, BD Pharmingen) or BV711 mouse anti-human CD49d (clone 9F10, BD Horizon) APC mouse anti-human CD54 (clone REA266, Miltenyi Biotec, Auburn, CA, USA), PE mouse anti-human CD106 (VCAM-1) (clone REA269, Miltenyi Biotec) or BV605 mouse anti-human CD106 (Clone 51-10C9, BD Horizon), FITC mouse anti-human CD44 (clone G44-26, BD Pharmingen) or APC mouse anti-human CD44 (clone G44-26) and PE CD184 (CXCR4) (clone 12G5, Miltenyi Biotec). Dead cells were excluded during acquisition and analysis. The FlowJo software (v10.0, FlowJo, LLC, Ashland, OR, USA) was used for data analysis.

### 4.12. Treatment with Cytotoxic Agents

Leukemic cells derived from patients (*n* = 5) that had shown a worse response at the final of the induction treatment were chosen for these experiments. B-ALL cells were treated during 6 h with 250 nM dexamethasone (Sigma-Aldrich, St. Louis, MO, USA) and then cells were co-cultured on 70% confluent MSC that have been pre-treated or not for 2 h with 40 µM HKPS, HK or 20 µM ENZA. Three days later, the unattached B-ALL cells were collected and the co-cultures were washed and trypsinized, and cells thus recovered were mixed with the corresponding unattached cells and double labelled with CD19 FITC (clone HIB19, BD Pharmingen, San Jose, CA, USA) and the LIVE/DEAD Fixable Aqua Dead Cell Stain (Molecular Probes, Eugene, OR, USA); evaluations and analysis were performed by flow cytometry (FACSAria IIIup Becton Dickinson Biosciences, San Jose, CA, USA) using the FlowJo software.

### 4.13. Statistical Analysis

Values were expressed as mean ± standard error of the mean (SEM) with *n* being the number of samples. Statistical significance was evaluated by non-parametric one-way analysis of variance (ANOVA) with Kruskal-Wallis test for viability experiments or when B-ALL cell adhesion was evaluated. The grouped analysis of all patient’s samples defining the ranges of response to HKPS treatment was made using ordinary One-way ANOVA. Comparisons between HKPS and STAU treatments in the HKPS most susceptible group were performed using Wilcoxon test and multivariate analysis using Pearson’s correlation. Correlation analysis was made employing the R studio software. The other analyses were performed using Prism 6.0 version software. *p* = 0.05 (*), *p* = 0.01 (**), *p* = 0.001 (***) and *p* = 0.0001 (****) were considered to be statistically significant.

## Supplementary Materials

Supplementary materials can be found at https://www.mdpi.com/1422-0067/21/10/3705/s1.

## Abbreviations

MSC	Mesenchymal stem cells
PKC	Protein kinase C
B-ALL	B-cell acute lymphoblastic leukaemia
CLL	Chronic lymphoid leukaemia
AML	Acute myeloid leukaemia
BM	Bone Marrow
MNC	Mononuclear cells
FBS	Fetal bovine serum
STAU	Staurosporine
ENZA	Enzastaurine
DEXA	Dexamethasone
NT	Non treated
TW	Transwell
MRD	Minimal residual disease
VCAM-1	Vascular cell-adhesion molecule-1
VLA	Very late antigen
