# Supplementary material for: Dual Targeting of Stromal Cell Support and Leukemic Cell Growth by a Peptidic PKC Inhibitor Shows Effectiveness against B-ALL
Source: Int J Mol Sci. 2020 May 25;21(10):3705. doi: 10.3390/ijms21103705 (PMC7279155; doi:10.3390/ijms21103705)
Supplement: Supplementary file 1 [file ijms-21-03705-s001.pdf]

SUPPLEMENTARY INFORMATION

Dual Targeting of Stromal Cell Support and Leukemic Cell Growth by a Peptidic PKC Inhibitor Shows Effectiveness against B-ALL

Paola Fernanda Ruiz-Aparicio[1], Natalia-Del Pilar Vanegas[1], Gloria Inés Uribe[2,3], Paola Ortiz-Montero[1], Camila Cadavid-Cortés[1], Jimmy Lagos[2], Jessica Flechas-Afanador[2], Adriana Linares-Ballesteros[2], Jean-Paul Vernot[1,4,*]

SAMPLE	AGE AT DIAGNOSIS	INFILTRATION (%)	HKPS RESPONSE GROUP	ALL TYPE	KARYOTYPE	INITIAL WBC	CNS INVOLVEMENT	RESPONSE AT DAY 8	RISK GROUP	REMISION AT DAY 15	MRD AT DAY 15	MRD AT THE END OF INDUCTION
B-ALL001	9	95%	III	COMMON	46,XX	43236	1	POOR	HIGH	NO	47%	40.40%
B-ALL002	10	92%	II	PRE-B	46,XX	38940	1	GOOD	INTERMEDIATE	YES	0%	0%
B-ALL003	2	93%	I	PRE-B	46,XY	4760	1	GOOD	HIGH	YES	85.50%	0%
B-ALL004	10	92%	I	PRE-B	46,XY	80750	1	GOOD	INTERMEDIATE	YES	0%	0%
B-ALL005	4	95%	III	PRE-B	46,XX	7550	1	GOOD	INTERMEDIATE	YES	1.27%	0%
B-ALL006	9	97%	III	PRE-B	46,XY	5140	1	GOOD	HIGH	YES	15.31%	0%
B-ALL007	17	80%	I	PRE-B	46,XX	10650	3	GOOD	HIGH	YES	21%	0%
B-ALL008	4	98%	III	COMMON	46,XY	4530	1	GOOD	INTERMEDIATE	YES	1%	0%
B-ALL009	4	98%	III	PRE-B	TRYSOMY 21	23190	1	GOOD	INTERMEDIATE	YES	3.47%	0%
B-ALL010	9	95%	II	COMMON	HYPERDIPLOID	8420	1	GOOD	INTERMEDIATE	YES	0.08%	0%
B-ALL011	2	95%	I	COMMON	HYPERDIPLOID	2740	1	GOOD	INTERMEDIATE	YES	4.70%	0%
B-ALL012	11	98%	II	PRE-B	46,XX	5300	3	GOOD	INTERMEDIATE	YES	0.04%	0%
B-ALL013	4	95%	I	COMMON	46,XY	6010	1	POOR	HIGH	NO	64%	0.76%
B-ALL014	4	89%	II	COMMON	46,XY	3360	1	GOOD	STANDARD	YES	0%	0%
B-ALL015	4	78%	II	COMMON	46,XY	12510	1	GOOD	HIGH	YES	13.53%	0.02%
B-ALL016	3	95%	III	COMMON	46,XX	7330	1	GOOD	INTERMEDIATE	YES	0.07%	0.02%
B-ALL017	3	90%	I	COMMON	46,XY	6920	1	POOR	INTERMEDIATE	YES	22.75%	0%
B-ALL018	6	89%	II	COMMON	HIPERDIPLOID	9710	1	POOR	HIGH	YES	3,90%	0%
B-ALL019	2	90%	III	COMMON	HIPERDIPLOID	20340	1	GOOD	INTERMEDIATE	YES	0,04%	0%
B-ALL020	5	85%	II	COMMON	HIPERDIPLOID	8100	1	GOOD	INTERMEDIATE	YES	1,44%	0%
B-ALL021	12	92%	II	COMMON	46, XY	8680	1	GOOD	INTERMEDIATE	YES	0%	0%
B-ALL022	2	98%	I	COMMON	46,XY	5888	1	GOOD	INTERMEDIATE	YES	4,37%	0%
B-ALL023	3	98%	II	COMMON	46,XX	24500	1	GOOD	INTERMEDIATE	YES	0%	0%

Table S1. B-ALL patient's characteristics, response to conventional therapy and effect of HKPS on isolated leukemic cells. 23 paediatric patients newly diagnosed with B-ALL with >80% blast infiltration were chosen for this study.

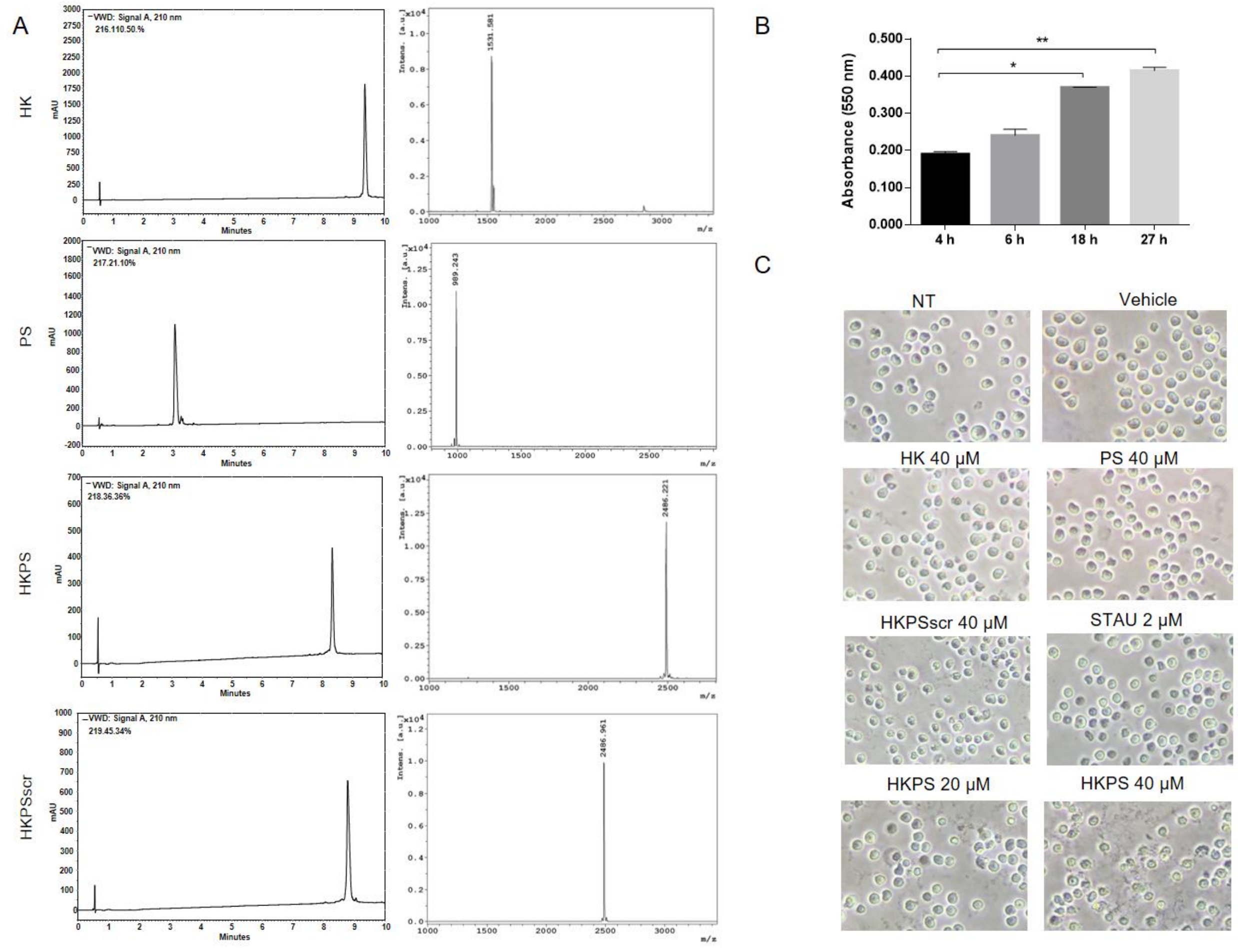

Figure S1. Peptides characterization, MTT assay in B-ALL and multiple correlations analysis. (**A**) Peptides were synthetized using the SPPS-Fmoc/tBu methodology. The purity was assessed by RP-HPLC and characterization was performed by MALDI-TOF mass spectrometry. (**B**) The formation of Formazan crystals in B-ALL cells was evaluated at different time points by MTT assay (p values: non-parametric one-way ANOVA *$p<0.05$. **$p<0.01$). (**C**) Morphological changes observed in a paediatric B-ALL patient sample after 2 h of treatment with HKPS, HK, PS, HKPSscr, STAU and vehicle at the indicated concentrations. NT = non-treated cells. Representative microphotographs are shown at 40x magnification.

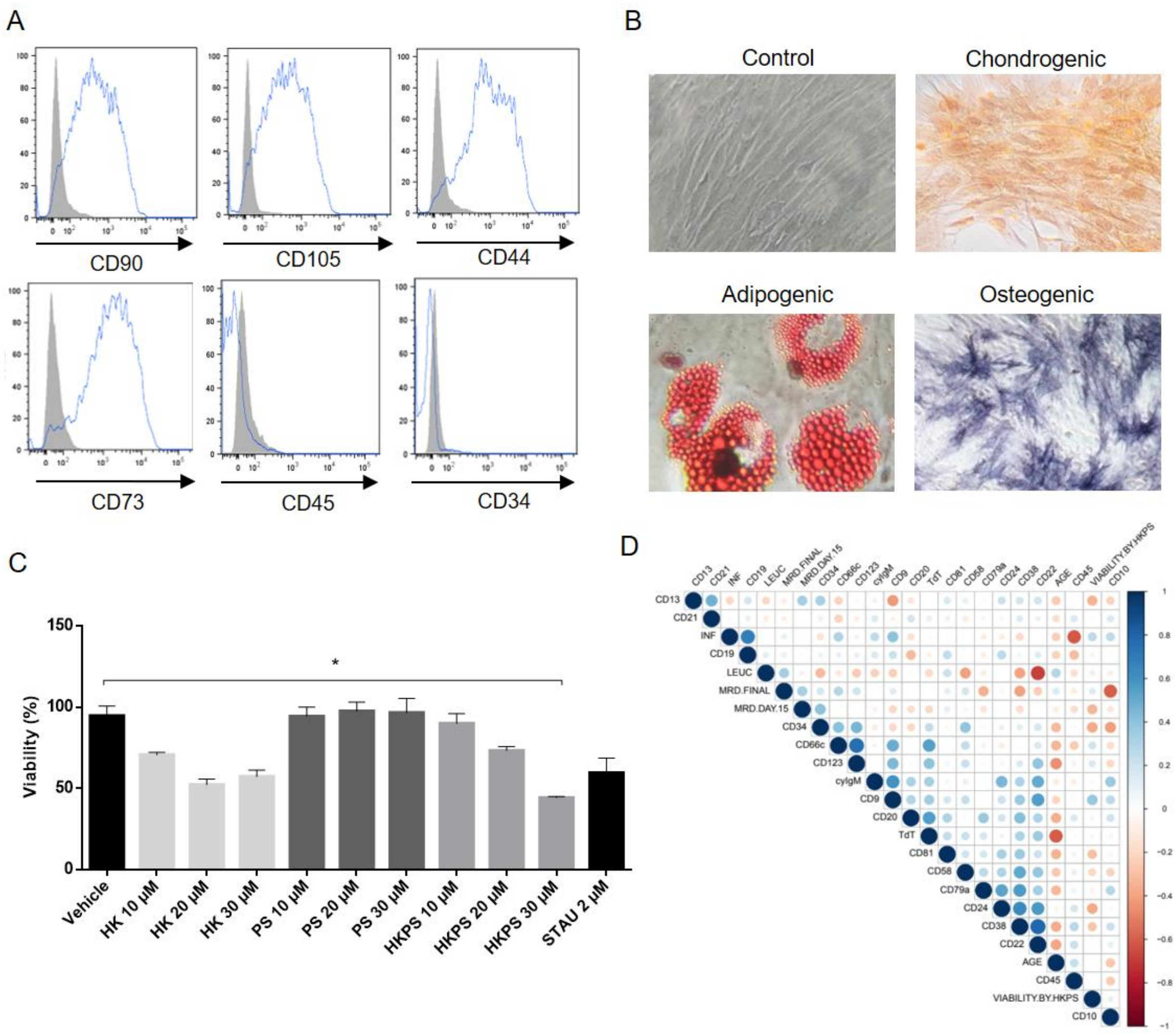

Figure S2. **Bone marrow MSC characterization, MSC viability after peptide treatment and multiple correlations analysis of B-ALL patient samples**. **(A)** MSC derived from orthopaedic paediatric patients were characterized by flow cytometry according to the CD90, CD105, CD44, CD73, CD45 and CD34 expression. Unstained MSC (grey histograms) were used as controls. (**B**) MSC were induced to differentiate for 21 days to the osteogenic lineage and for 14 days to chondrogenic and adipogenic lineages. After induction, differentiated cells were stained with Oil Red O (adipogenic), safranin O (chondrogenic) and using the NBT/BCIP Kit (osteogenic). Representative microphotographs are shown at the indicated magnifications. (**C**) MSC viability was assessed by MTT assay after treatment with peptides, STAU or controls for 6 h. Percentage of viability was determined in relation to non-treated MSC (**D**) Pearson's correlations were performed in 23 B-ALL patient samples. Patients' characteristics associated with immunophenotype, blasts infiltration (INF) at diagnosis and response after induction phase were compared with the B-ALL cells viability after treatment with HKPS. The size of the circles represents the strength and colours the direction of the linear relationship between variables. Data are expressed as mean ± SEM (p values: non parametric one-way ANOVA (C) and Pearson correlation (D) *$p<0.05$).

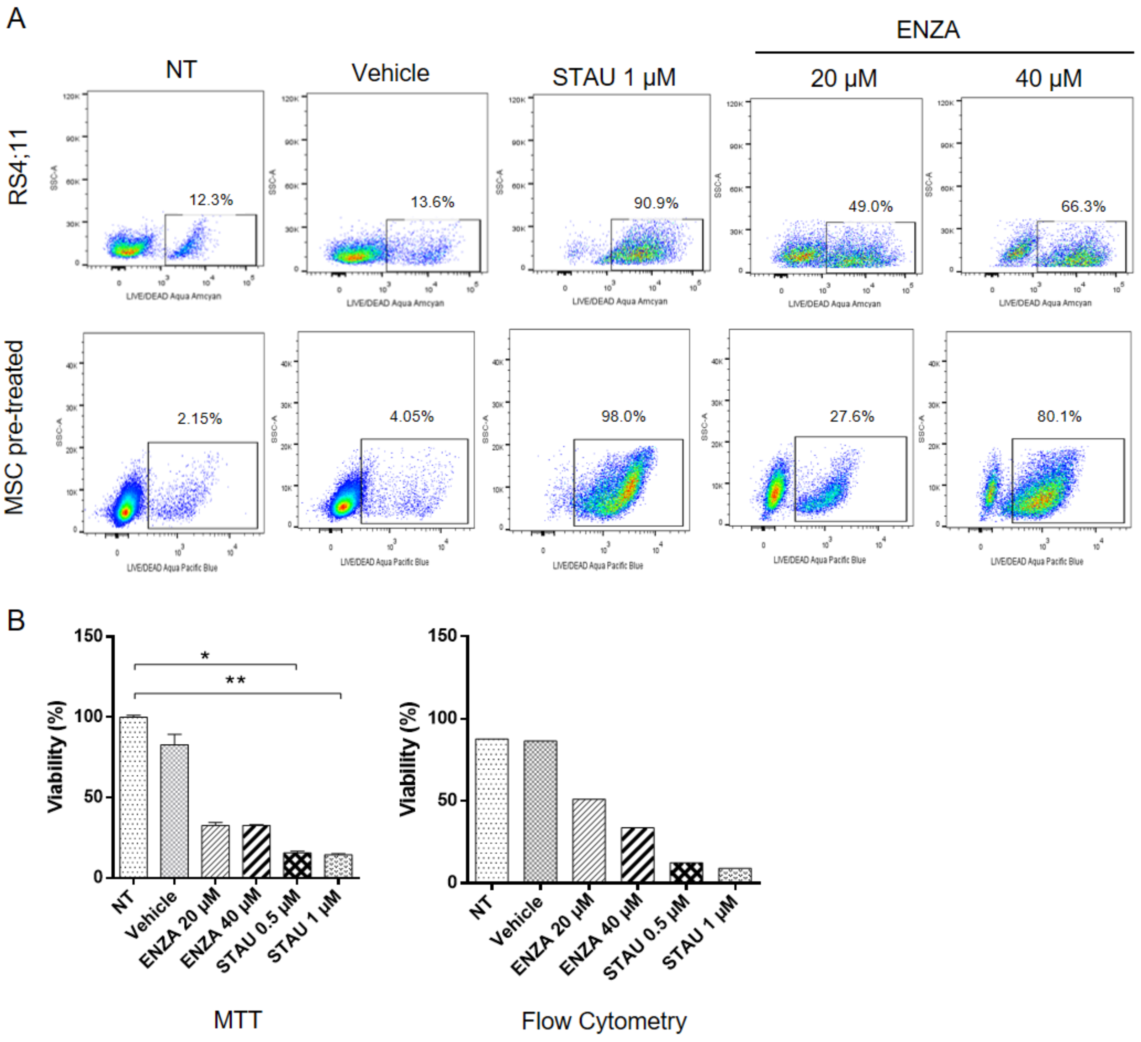

Figure S3. Treatment of the MSC support with PKC inhibitors impairs the survival of leukemic cells. (**A**) Effects on RS4;11 cell viability after direct treatment with PKC inhibitors ENZA and STAU for 24 h or after treatment of the MSC support for 24 h and further co-culturing with RS4;11 cells for additional 72 h. Cell viability in the leukemic cell population was determined by flow cytometry. Percentages in squares correspond to RS4;11 cells positive for LIVE/DEAD Aqua staining (dead cells). The gate was defined with unlabelled leukemic cells alone or co-cultures. (**B**) Comparison of the flow cytometry and MTT assays. Data are expressed as mean ± SEM (p values: non parametric one-way ANOVA *p<0.05, **p<0.01).

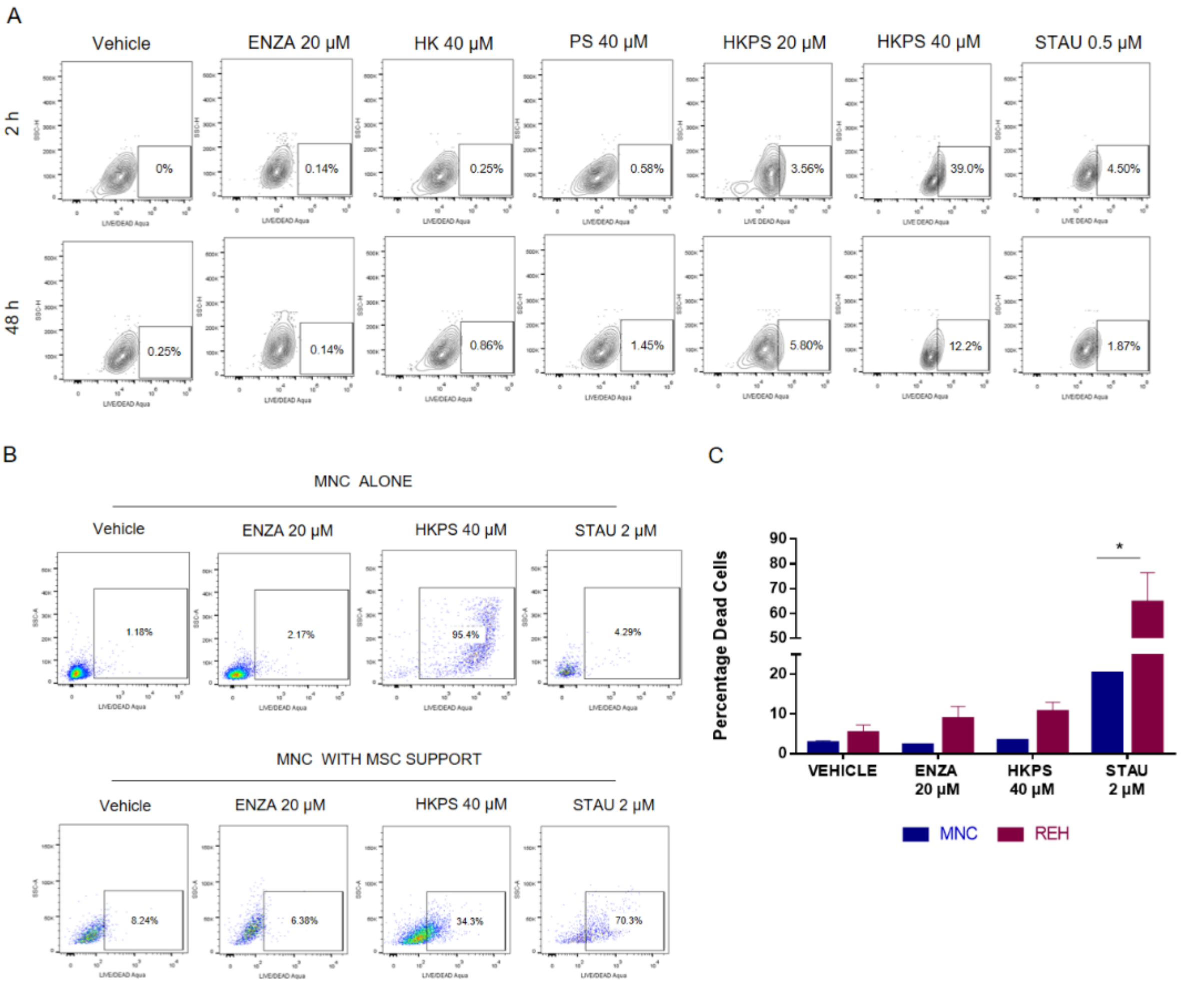

Figure S4. HKPS treatment slightly affects the viability of MSC and healthy MNC. (A) MSC viability was determined by flow cytometry after treatment with peptides or STAU and ENZA inhibitors for 2 h and 48 h in the presence of 1% FBS, as indicated. After treatments, B-ALL cells were co-cultured with MSC for 72 h. Co-cultures were collected and double labelled with CD105 and LIVE/DEAD Aqua to distinguish the specific effect on MSC viability. Percentages in squares correspond to CD105 and LIVE/DEAD Aqua positive MSC. The gate was defined with unlabelled co-cultures. A representative experiment of two is shown. (**B**) Viability of healthy MNC was assessed employing the LIVE/DEAD aqua staining after 2 h of treatment with the different compounds. The role of the mesenchymal support was evaluated on healthy MNC viability after MSC pre-treatment with ENZA, HKPS or STAU for 2 h and additional 72 h of co-culture. (**C**) MNC obtained from healthy donors and REH cells in a 1:1 proportion were co-cultured with pre-treated MSC (2 h) with HKPS, ENZA or STAU at the indicated concentrations and then cell viability was evaluated by flow cytometry after 72 h. The gates were defined with unlabelled cell populations. Data are expressed as mean ± SEM (p values: non parametric one-way ANOVA *$p<0.05$).

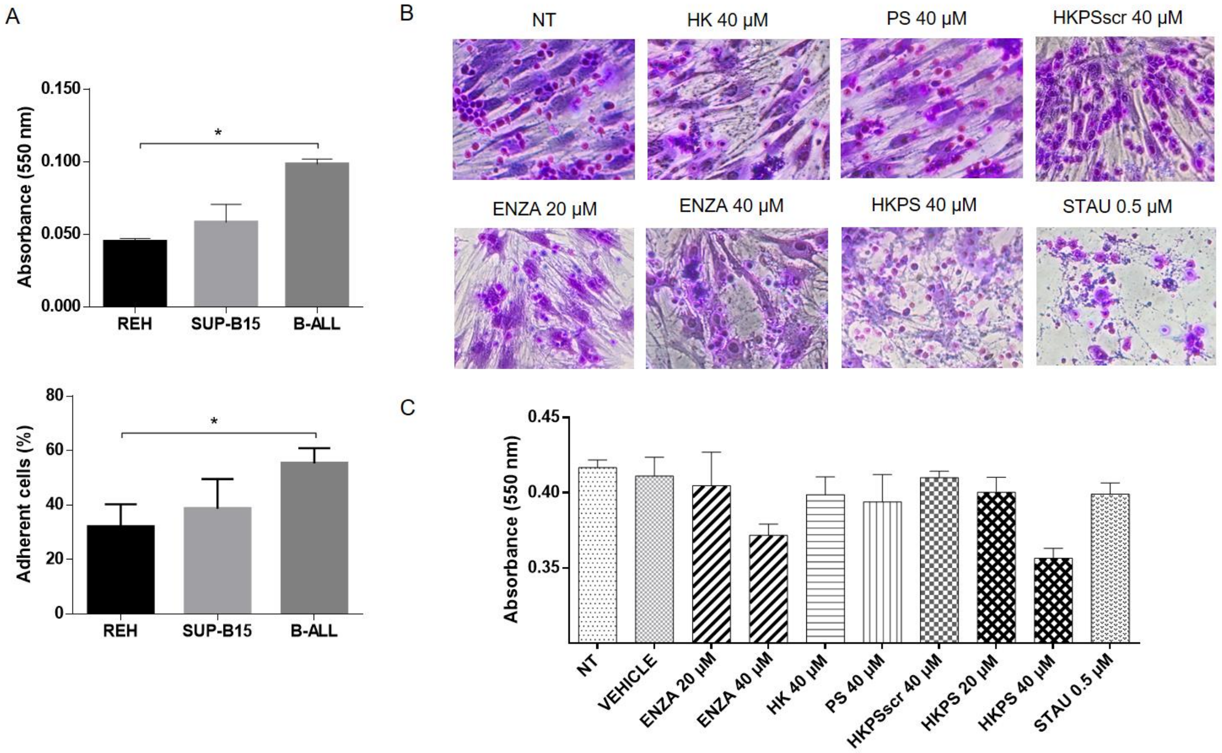

Figure S5. B-ALL cells from patients exhibit a higher adhesion to MSC support compared to leukemic cell lines, with HKPS affecting this capacity. (**A**) The leukemic cell lines REH and SUP-B15, and a B-ALL patient sample were co-cultured with MSC and the adhesion capacity was evaluated after 6 h. Non-adherent leukemic cells were harvested by washing with PBS and then the adherent cells were stained with crystal violet. Adhesion of B-ALL cells was determined indirectly by absorbance after dissolving the stain with PBS-EDTA (upper panel) or directly, after counting the non-adherent B-ALL cells recovered from the washing procedure (lower panel). (**B**) MSC were pre-treated 2 h with peptides, ENZA or STAU at the indicated concentrations and the adhesion assay using B-ALL patient cells were performed after 6 h, as described before. Representative images at 20x magnification of the adherent cells in the co-cultures after MSC treatments and removal of unattached cells are shown. (**C**) Absorbance values measured after staining with crystal violet. Data are expressed as mean ± SEM (p values: non parametric one-way ANOVA $^*p<0.05$).

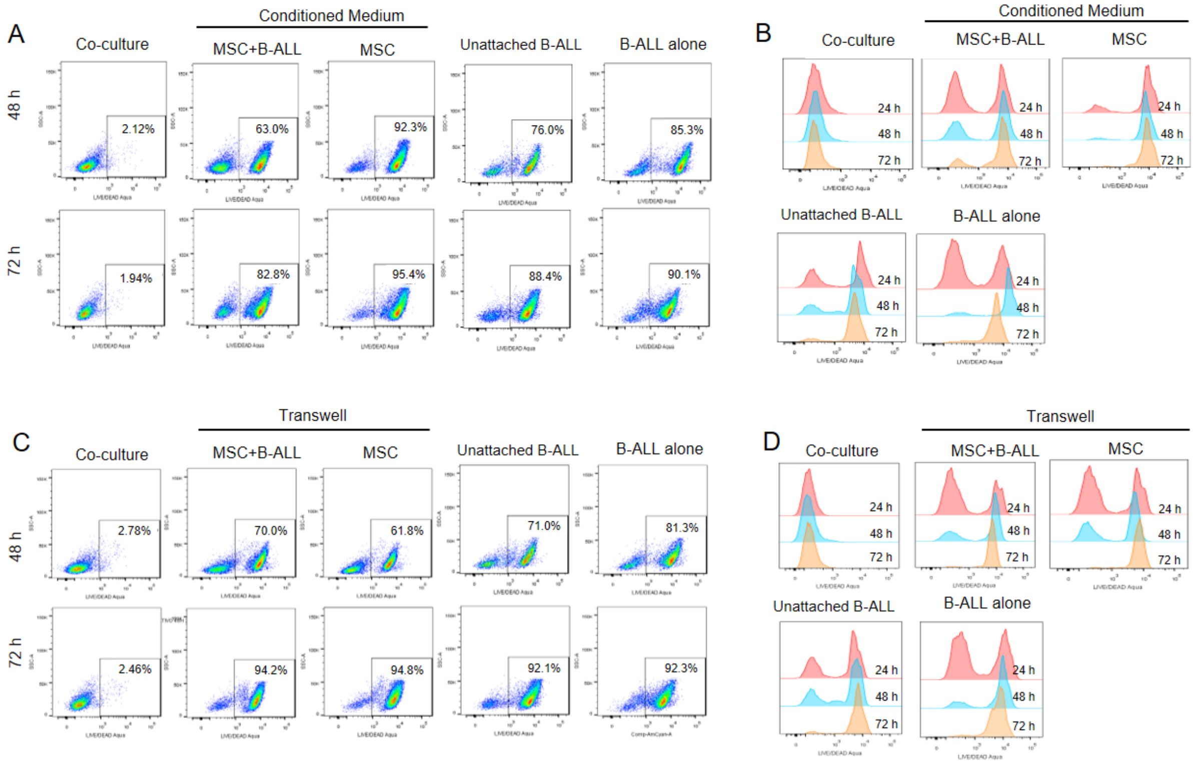

Figure S6. In the absence of MSC, B-ALL cells show high percentages of dead cells, with soluble factors contributing to a lesser extend to leukemic cells survival. (A) B-ALL cells viability was assessed by flow cytometry after incubation with fresh conditioned medium from co-cultures or MSC alone, for 48 and 72 h. A refeeding with fresh media was made at 36 h. Controls of leukemic cells in direct contact with MSC support and B-ALL cells alone were included. (**B**) Comparison of living and dead B- ALL cells in the different conditions in A. (**C**) Survival of B-ALL cells in the presence of soluble factors produced by co-cultures or MSC alone in a Transwell system. B-ALL cells alone or B-ALL cells and MSC in direct contact were used as controls. Percentages in squares correspond to CD19+ B-ALL cells positive for LIVE/DEAD Aqua staining. The gate was defined with unlabelled co-cultures. (**D**) Comparison of living and dead B-ALL cells in the different conditions in C.

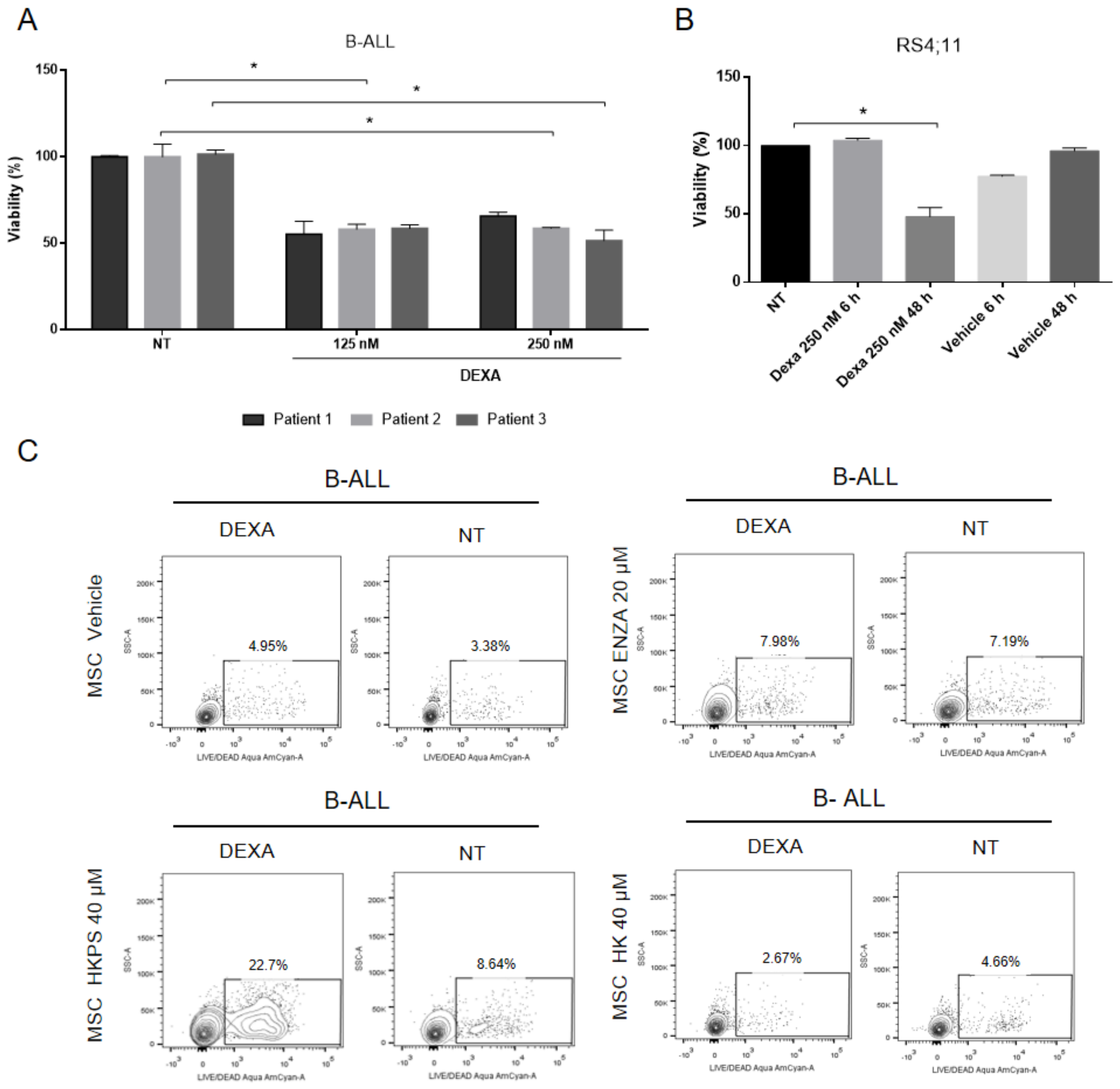

Figure S7. Susceptibility of B-ALL cells to DEXA in the presence or absence of MSC treated or not with HKPS. (**A**) Three different patient cell samples were treated with DEXA at the indicated concentrations for 6 h. The cell viability was evaluated by the MTT assay. (**B**) RS4;11 cells were treated during 6 h with DEXA 250 nM and then cell viability was evaluated at the end of the treatment period and after 42 h of further incubation without treatment. Cytotoxic effect of DEXA was determined by the MTT assay. **(C)** MSC were pre-treated with HKPS and HK peptides (40 µM), ENZA (20 µM) or vehicle for 48 h in the presence of 1% FBS. Next, B-ALL cells treated or not (NT) to DEXA (250 nM) were co-cultured with the pre-treated MSC. The viability of B-ALL cells was evaluated by flow cytometry 72 h after establishment of the co-cultures. Percentages in squares correspond to CD19+ B-ALL cells positive for LIVE/DEAD Aqua staining. The gate was defined with unlabelled co-cultures. Data are expressed as mean ± SEM (p values: non parametric one-way ANOVA *p<0.05).
